# Baseline characteristics of patients with chronic kidney disease stage 3 and stage 4 in spain: the MERENA observational cohort study

**DOI:** 10.1186/1471-2369-12-53

**Published:** 2011-10-05

**Authors:** Alberto Martínez-Castelao, José L Górriz, José M Portolés, Fernando De Alvaro, Aleix Cases, José Luño, Juan F Navarro-González, Rafael Montes, Juan J De la Cruz-Troca, Aparna Natarajan, Daniel Batlle

**Affiliations:** 1 Nephrology Unit, Hospital Universitario Bellvitge, L'Hospitalet de Llobregat, Barcelona, Spain; 2 Nephrology Unit, Hospital Universitario Dr. Peset, Valencia, Spain; 3 Nephrology Unit, Fundación Hospital Alcorcón, Madrid, Spain; 4 Nephrology Unit, Hospital Universitario La Paz, Madrid, Spain; 5 Nephrology Unit, Hospital Clínic, Universitat de Barcelona, Barcelona, Spain; 6 Nephrology Unit, Hospital General Universitario Gregorio Marañón, Madrid, Spain; 7 Nephrology Unit, Hospital Universitario Nuestra Señora de la Candelaria, Santa Cruz de Tenerife, Spain; 8 Nephrology Unit, Hospital Virgen del Rocío, Sevilla, Spain; 9 Department of Preventive Medicine and Public Health, Universidad Autónoma, Madrid, Spain; 10 Northwestern University Feinbeg School of Medicine, Chicago, USA

## Abstract

**Background:**

To obtain information on cardiovascular morbidity, hypertension control, anemia and mineral metabolism based on the analysis of the baseline characteristics of a large cohort of Spanish patients enrolled in an ongoing prospective, observational, multicenter study of patients with stages 3 and 4 chronic kidney diseases (CKD).

**Methods:**

Multicenter study from Spanish government hospital-based Nephrology outpatient clinics involving 1129 patients with CKD stages 3 (n = 434) and 4 (n = 695) defined by GFR calculated by the MDRD formula. Additional analysis was performed with GFR calculated using the CKD-EPI and Cockcroft-Gault formula.

**Results:**

In the cohort as a whole, median age 70.9 years, morbidity from all cardiovascular disease (CVD) was very high (39.1%). In CKD stage 4, CVD prevalence was higher than in stage 3 (42.2 vs 35.6% p < 0.024). Subdividing stage 3 in 3a and 3b and after adjusting for age, CVD increased with declining GFR with the hierarchy (stage 3a < stage 3b < stage 4) when calculated by CKD-EPI (31.8, 35.4, 42.1%, p 0.039) and Cockcroft-Gault formula (30.9, 35.6, 43.4%, p 0.010) and MDRD formula (32.5, 36.2, 42.2%,) but with the latter, it did not reach statistical significance (p 0.882). Hypertension was almost universal among those with stages 3 and 4 CKD (91.2% and 94.1%, respectively) despite the use of more than 3 anti-hypertensive agents including widespread use of RAS blockers. Proteinuria (> 300 mg/day) was present in more than 60% of patients and there was no significant differences between stages 3 and 4 CKD (1.2 ± 1.8 and 1.3 ± 1.8 g/day, respectively). A majority of the patients had hemoglobin levels greater than 11 g/dL (91.1 and 85.5% in stages 3 and 4 CKD respectively p < 0.001) while the use of erythropoiesis-stimulating agents (ESA) was limited to 16 and 34.1% in stages 3 and 4 CKD respectively. Intact parathyroid hormone (i-PTH) was elevated in stage 3 and stage 4 CKD patients (121 ± 99 and 166 ± 125 pg/mL p 0.001) despite good control of calcium-phosphorus levels.

**Conclusion:**

This study provides an overview of key clinical parameters in patients with CKD Stages 3 and 4 where delivery or care was largely by nephrologists working in a network of hospital-based clinics of the Spanish National Healthcare System.

## Background

Although progress has been made in retarding the progression of chronic kidney disease (CKD) to end-stage renal disease (ESRD), the prevalence of CKD as a whole is rising in the United States and worldwide [1-5]. CKD is associated with increased morbidity and mortality, a negative impact on quality of life, progression to ESRD, and represents an important burden for the healthcare system [3,4]. In the CKD population, the risk of death and cardiovascular events increases proportionally with the decline of renal function, the risk of mortality exceeding that seen in ESRD patients requiring renal replacement therapy (RRT) in some studies [6-11]. According to data from the 1999-2004 National Health and Examination Survey (NHANES) [1], the crude CKD prevalence estimate for adults' aged 20 years or older in the United States was 16.8%. Two large epidemiological studies carried out in Spain showed a variable prevalence of CKD, ranging from 7.7% in the general population [12] to 21.3% in a large cohort of patients attended by primary care physicians [13].

The National Kidney Foundation's Kidney Disease Outcomes Quality Initiative (K/DOQI) guidelines [14] represent an important tool to evaluate patients with different CKD stages. Because of the high prevalence of co morbidities in CKD patients, achieving guideline-based targets may be difficult. In fact, a cross-sectional study reported that the treatment of modifiable cardiovascular risk factors in pre-dialysis CKD patients was largely inadequate [14]. Data from large prospective observational studies of the clinical course of patients with CKD, particularly regarding morbidity and mortality and achievement of targets based on guidelines are scarce [15-17]. We wanted to obtain information relative to not only cardiovascular morbidity but also data relative to hypertension, anemia and mineral metabolism from a large and contemporary Spanish cohort called *Morbimortalidad en Enfermedad REnal en pacieNtes diAbéticos y no diabéticos *(MERENA), which translates into Morbimortality in CKD Stage 3-4 in Diabetic and Non-diabetic Patients. MERENA is an ongoing prospective, observational, multicenter study aimed to assess outcomes, such as renal disease progression, as well as morbidity and mortality in a cohort of CKD stages 3-4, with a planned follow-up of 5 years. Here we report the baseline characteristics of this cohort, a total of 1,129 patients which provide a timely overview of the status of CKD care delivery for Stages 3 and 4. For the purposes of this report, we did not analyze potential differences between diabetic and non-diabetic subjects, an objective of the MERENA study; rather here we choose to direct our focus towards the analysis of CKD by stages regardless of diabetes status.

## Methods

### Materials and methods

The MERENA study is a prospective, observational, non-intervening, multi-center study designed by the Spanish Study Group for Diabetic Nephropathy *Grupo Español de Estudio de la ****N****efropatía DIABética *(GEENDIAB) of the Spanish Society of Nephrology to characterize the clinical management and outcomes after a 5-year follow-up of two cohorts, diabetic and non-diabetic, of patients with CKD stages 3-4 in a clinical practice setting. Secondary objectives included the assessment of cardiovascular comorbidities, blood pressure (BP), glycemic and lipid control, use of antiplatelet therapy, management of anemia and CKD-MBD, and degree of adherence to clinical practice guidelines by attending nephrologists. The study was conducted at the outpatient nephrology clinics of 55 hospitals evenly distributed through the Spanish territory from the Spanish National Healthcare system with free and equal access to all citizens.

### Study Population

All patients older than 18 years seen at participating nephrology units with CKD stages 3 and 4 (using MDRD-4 equation) according to K/DOQI guidelines [14] from December 1, 2003 to April 30, 2004 were eligible for inclusion in the study, whether they were incident (newly referred) or prevalent patients. Exclusion criteria were as follows: age < 18 yr, CKD stages other than 3 or 4, estimated time to progression to ESRD < 12 months, evidence of consumptive or disabling disease, malignancy, and active infection, inflammatory disorders, or expected death within 12 months. All participants gave written informed consent. The Ethics Committee of the Hospital Universitario Bellvitge in Barcelona approved the study protocol.

### Assessment and Data Collection

All consecutive patients who met the inclusion criteria of the study during the enrolment period were included. Standardized data collection was ensured and conducted by nephrologists using internet-based electronic case report forms. Baseline data included: sex, age, weight, height, body mass index (BMI), specialty of the physician who referred the patient to the nephrologist, K/DOQI CKD stage, and associated co morbid conditions, such as cardiovascular disease, diabetic status, hyperlipidemia, hypertension, or smoking status. The Charlson co-morbidity index was calculated. Renal function was estimated from the 4-variable Modification of Diet in Renal Disease (MDRD) equation, CKD-EPI and Cockroft-Gault equations were also used.

All the participating centers used the same criteria to define existing co morbidities and pathologies. These had been defined in the study's protocol, and together with the recommendations for adherence to the clinical practice guidelines, they had been published on the researchers' website.

Criteria for the diagnosis of coronary artery disease included history of acute myocardial infarction, angina or revascularization procedure. Cerebrovascular disease included transient ischemic attack, stroke, and hemiplegia. Peripheral vascular disease included lower limb intermittent claudication, revascularization (surgical or percutaneous) or amputation. Cardiovascular disease was defined as the history of at least one of the following: ischemic heart disease, cerebrovascular disease and peripheral vascular disease. Cardiac disease was defined as the history of one of the following: coronary artery disease, myocardial infarction, congestive heart failure or history of coronary revascularization. All diabetic patients met the classification criteria established by the American Diabetes Association. The diagnosis of diabetic nephropathy as a likely cause of CKD was made by each investigator. This was generally based on the presence of retinopathy, proteinuria diabetes mellitus duration and all relevant clinical data. Hypertension was considered if the patient had a BP ≥ 140/90 mm Hg or need for antihypertensive drugs. Dyslipidemia included total serum cholesterol > 5.2 mmol/L (200 mg/dL), or triglycerides > 1.7 mmol/L (150 mg/dL), or high-density lipoprotein (HDL) cholesterol < 1.0 mmol/L (40 mg/dL) in males or 1.3 mmol/L (48 mg/dL) in females or low-density lipoprotein (LDL) cholesterol > 2.6 mmol/L (100 mg/dL) or need of lipid-lowering drugs. Drugs prescribed were also recorded. Anemia was defined according to the K/DOQI 2006 Guidelines (serum hemoglobin < 12 g/dL in females and 13.5 g/dL in males) and by the 2004 revised European Best Practice Guidelines (EBPG) [18] (serum hemoglobin < 11.5 g/dL in females and 13.5 g/dL in males, and 12.0 g/dL in men aged > 70 years) or use of erythropoiesis-stimulating agents (ESA) at any Hb level.

Blood samples were analyzed at the respective hospital laboratories of the participating centers. Complete blood cell count, glycated hemoglobin (HbA1c), serum albumin and proteins, glucose, urea, creatinine, uric acid, lipid profile, iron status, potassium, bicarbonate, calcium, phosphorus, intact parathyroid hormone (i-PTH), and 24-hour urinalysis (proteinuria, creatinine, and urea nitrogen) were determined.

Missing data of the main variables at baseline are noted in brackets as follows: MDRD equation (0%), blood pressure (0%), triglycerides (3%), HDL-c (7.1%), LDL-c (7.1%), HbA1c in diabetics (15%), hemoglobin (0%), serum ferritin (7.4%), TSAT (31%), calcium (2%), phosphorus (2%), and i-PTH (12.7%).

The referring physicians for the entire study included: primary care physicians in 46.2% of cases, internal medicine in 12%, endocrinology in 8.9%, urology in 6.9%, and cardiology in 4.8%; the remaining patients were referred by other specialists or after a hospital admission.

Each investigator was advised to adhere strictly to the best practice guidelines and clinical recommendations concerning: lifestyle practices, target BP, HbA1c in diabetics and lipid levels, management of anemia and bone mineral disorders or use of antiplatelet agents. Recommendations were uploaded onto the study's researchers' website. The STROBE statement checklist has been considered in data analysis and in the preparation of the manuscript.

### Statistical Analysis

Data are presented as numbers and percentages, or as mean and standard deviation (SD). Differences between diabetic and non-diabetic patients were assessed with Student's *t *test for quantitative variables and the chi-square (χ^2^) test for categorical variables. Statistical significance was set at *P *< 0.05. Variables significantly associated with cardiovascular disease were assessed using binary logistic regression with forward stepwise selection procedure. Categorical variables were also adjusted by age when indicated. Analysis was performed using SPSS version 15.0 for Windows.

## Results

### General characteristics

The mean age of the total cohort of 1,129 patients was 68 ± 13 years, (median age 70.9, range 19-94 years). There were no significant differences in age between CKD stages 3 and 4 (Table 1). There was a predominance of male subjects (64%) and the ethnicity was all Caucasian. The subjects had been follow up in Nephrology clinics for 24 months (24 ± 12) at the point of entry into the study although it varied widely within the cohort (range: 0-46 months). Only 70 patients (6.2%) were truly incidents (new patients). The mean BMI was 28.4 kg/m^2 ^and 31% of the patients had BMI exceeding 30 kg/m^2^. BMI was significantly higher in stage 4 than stage 3 CKD (Table 1) BMI in male was lower than in female (28.0 ± 4.3 vs. 29.4 ± 5.8. p < 0.001).

**Table 1:** General characteristics and renal parameters:

Characteristics	Total (n = 1129)	CKD Stage 3 (*n *= 434)	CKD Stage 4 (*n *= 695)	*P *value
Age (yr; mean ± SD]) [range]	68 ± 13 [19-94]	68 ± 13 [19-89]	67 ± 13 [19-94]	0.428

Male gender (%)	64.0	75.3	57.0	0.001

Female gender (%)	36.0	24.7	43.0	< 0.001

Previous follow-up (months) [range]	24 ± 12 [0-46]	25 ± 7 [0-46]	23 ± 4 [0-46]	0.004

BMI (kg/m^2^; mean ± SD) [range]	28.4 ± 4.9 [15.6-60]	27.8 ± 4.3 [15.6-60]	29.0 ± 5.2 [18.2-43]	< 0.001

Patients with BMI > 30 kg/m^2 ^(%)	31.7	24.5	36.1	0.001

Patients with BMI between 25-29.9 kg/m^2 ^(%)	45.7	49.0	43.6	0.096

Patients with BMI < 25 kg/m^2 ^(%)	22.7	26.4	20.3	0.018

Diabetes Mellitus (%)	40.8	46.1	37.6	0.003

Serum creatinine (mg/dL) [range]	2.4 ± 0.7 [1.3-5.7]	2.0 ± 0.5 [1.3-3.5]	2.7 ± 0.6 [1.7-5.7]	0.001

eGFR (MDRD) ml/min/1.73 m2 [range]	28 ± 8 [15-58]	33 ± 8 [30-58]	23 ± 5 [15-29]	0.001

Cockcroft-Gault creat. clear. ml/min [range]	30 ± 8 [12-62]	37 ± 7 [21-62]	23 ± 4 [12-4]	0.001

There was also a high percentage of individuals with history of diabetes mellitus (40.8%) (n = 461) (91.7% type 2 diabetes mellitus and 8.2% type 1 diabetes mellitus), but diabetes as the cause of kidney disease was only attributed to 26.1% of the total cohort based on clinical assessment (See methods). The other causes of CKD were: vascular disease (in 28% of patients), glomerular disease (11.4%), interstitial renal disease (10.8%), polycystic kidney disease (3.5%), and others (5.7%), and the cause of the remaining 14.5% of cases were unknown.

The mean creatinine of the cohort was 2.4 mg/dL. The estimated creatinine clearance was 30 ± 8 ml/min when calculated by the Cockcroft-Gault formula. The MDRD estimated GFR was 28.8 ml/min/1.73 m^2^. Using the MDRD formula for CKD staging, we found 38.5% of subjects with stage 3 CKD and 61.5% of subjects with stage 4 CKD.

The percentage of CKD stage 4 patients with BMI > 30 kg/m^2 ^was significantly higher than in CKD stage 3 patients (36.1 vs. 24.5%) despite the percentage of individuals with diabetes mellitus being lower in CKD stage 4 as compared with CKD stage 3 (37.6 vs. 46.1%).

### Cardiovascular morbidity

History of cardiovascular disease (CVD) was reported in 39.1% of the cohort (Table 2). The percentage of patients with history of congestive heart failure was 17.5% of the total cohort. History of coronary artery disease was present in 21.6% with a prevalence of history of myocardial infarction in 11.1% of the patients. Cerebrovascular disease was documented in 12.2% and peripheral vascular disease in 19.7% of the patients. History of current smoking was elicited in 9.6% of the patient cohort while 36.2% of the patients were former smokers.

**Table 2:** Cardiovascular morbidity

Characteristics	Total (n = 1129)	CKD Stage 3 (*n *= 434)	CKD Stage 4 (*n *= 695)	*P *value
Cardiovascular disease (CVD) (%)^a^	39.1	35.6	42.2	0.024

Percentage of patients < 60years with CVD	19.8	19.2	20.1	NS

Percentage of patients > 60years with CVD	45.6	47.1	44.5	NS

Cardiac disease (%)^b^	35.1	31.3	38.5	0.012

Congestive heart failure (%)	17.5	15.1	19.7	0.039

Coronary artery disease (%)	21.6	20.7	22.4	0.362

Myocardial Infarction (%)	11.1	11.0	11.2	0.958

Cerebrovascular disease (%)	12.2	10.4	13.7	0.050

Peripheral vascular disease (%)	19.7	18.7	20.0	0.289

Current smoking (%)	9.6	10.5	8.8	0.410

Former smokers (%)	36.2	35.7	36.7	0.525

^a ^cardiovascular disease defined as the history of at least one of the following: ischemic heart disease, cerebrovascular disease and peripheral vascular disease.

^b ^cardiac disease defined as the history of one of the following: coronary artery disease, myocardial infarction, congestive heart failure or history of coronary revascularization

CKD stage 4 patients had a significantly higher percentage of CVD than CKD stage 3 patients (42.2 vs. 35.6%) (Table 2). The percentage of CVD was much higher in older subjects (age ≥ 60yrs) than in younger subjects (age < 60 yrs) (45.6 vs. 19.8%, p = 0.001).

The history of cardiac disease was also significantly higher in Stage 4 CKD than in stage 3 CKD (38.5 vs. 31.3% p < 0.01). There was also a significantly higher history of congestive heart failure (CHF) in stage 4 and 3 CKD patients (19.7% vs. 15.1%, p < 0.045) (Table 2). There were no significant differences in coronary artery disease, myocardial infarction, peripheral vascular disease and cerebrovascular disease between stage 4 CKD and stage 3 CKD patients (table 2).

**Figure 1:**
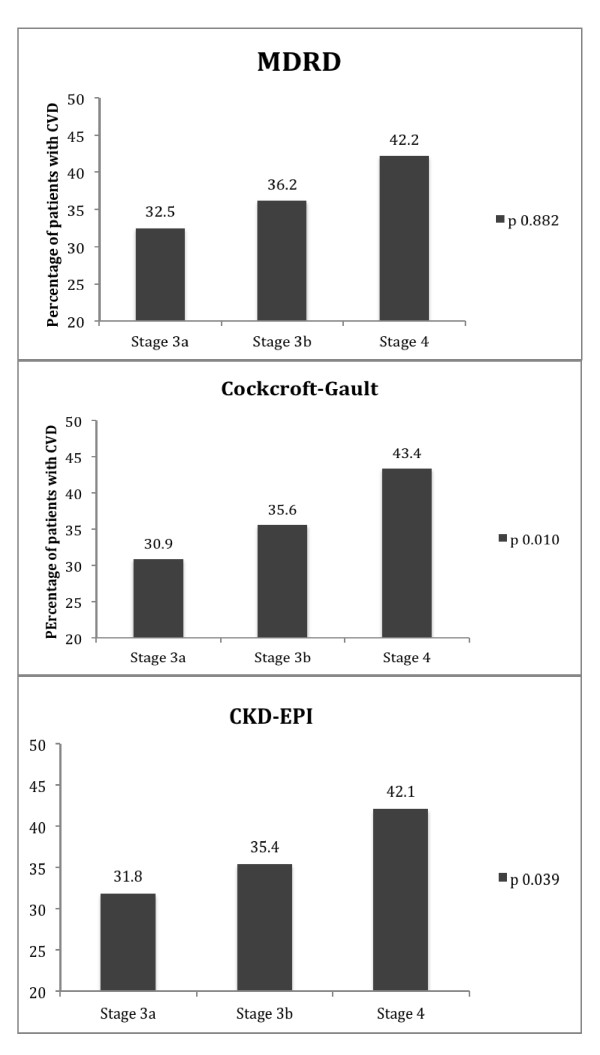
**Overall cardiovascular disease (CVD)**. An increase in CVD is shown as GFR declines using MDRD formula, upper panel, Cockcroft-Gault formula middle panel, and EPI-CKD formula lower panel. The increase in CVD is statistically significant using the Cockcroft-Gault, and EPI-CKD formula but not with MDRD formula.

To better examine CVD as a function of estimated GFR and given the wide rage of GFR in stage 3, we used the staging of 3a and 3b for this analysis. After adjusting for age, CVD increased with declining GFR with the hierarchy (stage 3a < stage 3b < stage 4) when calculated by CKD-EPI (31.8, 35.4, 42.1%, p 0.039) and Cockcroft-Gault formula (30.9, 35.6, 43.4%, p 0.010) and MDRD formula (32.5, 36.2, 42.2%,) but with the latter, it did not reach statistical significance (p 0.882) (Figure 1).

**Figure 2:**
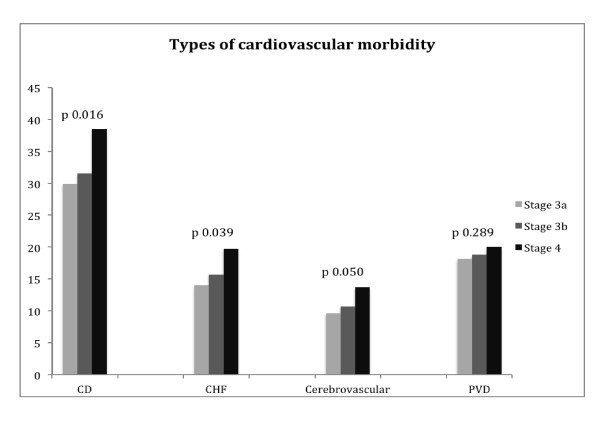
**Summary of cardiovascular morbidity types based on staging of CKD (stages 3a, 3b, 4)**. A significant increase in cardiac disease (CD), congestive heart failure (CHF) and cerebrovascular disease is seen. The increase in Peripheral vascular disease ( PVD) was not statistically significant.

After subdividing stage 3 CKD into stages 3a and 3b, there were also significant differences in age adjusted cardiac disease, congestive heart failure and cerebrovascular disease but not peripheral vascular disease (Figure 2).

### Blood pressure, proteinuria and use of anti-hypertensive agents

A majority of subjects had history of hypertension (92.7%) and there was no significant difference in the prevalence of hypertension between CKD stages 3 and 4 (Table 3). The definition of hypertension included subjects on antihypertensive treatment (See methods).

**Table 3:** Blood pressure, proteinuria and use of anti hypertensive agents:

Characteristics	Total (n = 1129)	CKD Stage 3	CKD Stage 4	*P *value
Hypertension (%)	92.7	91.2	94.1	0.06

Systolic BP < 130 mm Hg (%)	22.1	22.5	21.6	0.72

Diastolic BP < 80 mm Hg (%)	51.4	46.4	55.9	0.001

% of patients with BP < 130/80 mm Hg	17.4	19.4	16.3	0.18

% of patients with BP < 140/90 mm Hg	40.8	45.9	37.7	0.007

Systolic BP (mm Hg; mean ± SD)	141 ± 19	141 ± 19	142 ± 20	0.4

Diastolic BP (mm Hg; mean ± SD)	76 ± 11	78 ± 10	75 ± 11	0.001

Percentage of pts with proteinuria (> 300 mg/24 hrs) (%)	62.8	56.6	66.8	0.001

Proteinuria (g/24 h; mean [SD])	1.2 ± 1.8	1.2 ± 1.8	1.3 ± 1.8	0.60

Angiotensin-converting enzyme (ACE) inhibitors (%)	43.3	46.9	40.0	0.02

Angiotensin II receptor blockers (ARB) (%)	44.8	47.7	42.2	0.06

Combined ACE and ARB (%)	10.8	12.1	9.6	0.18

Diuretics (%)	65.7	61.5	69.6	0.004

Calcium channel blockers (%)	45.7	45.8	45.6	0.94

Beta blockers (%)	21.1	22.5	19.8	0.25

Alpha blockers (%)	23.4	23.5	23.3	0.95

% of patients with 3 or more antihypertensive drugs	47.7	45.8	49.7	0.18

Mean serum potassium levels (mEq/dL; mean ± SD, [range])	4.82 ± 0.60 [3-6.7]	4.71 ± 0.57 [3-6.4]	4.88 ± 0.61 [3.1-6.7]	0.001

% of patients with potassium levels > 5.4 mEq/dL	14.3	9.8	17.1	0.001

The percentage of patients with a systolic BP < 130 mm Hg and a diastolic BP < 80 mm Hg was 22.1% and 51.4%, respectively. Only 17.4% of the total cohort had systolic BP < 130 mm Hg and diastolic BP < 80 mm Hg (Table 3).

For the cohort as a group, the mean systolic BP was 141 ± 19 mm Hg and mean diastolic BP was 76 ± 11 mm Hg. CKD Stage 3 and 4 patients had a mean systolic BP which was not significantly different from each other. The mean diastolic BP, however, was slightly but significantly higher in stage 3 than stage 4 patients.

The percentage of patients on 3 or more anti-hypertensive drugs for the total cohort was 47.7% and there were no significant differences between stage 3 and 4 (Table 3). A slightly higher percentage of patients were on 3 or more anti-hypertensive medications in stage 4 than stage 3 CKD but the difference was not significant. The percentage of patients on angiotensin-converting enzyme (ACE) inhibitors was 43.3% and on Angiotensin II receptor blockers (ARB) was 44.8%. The patients on combination therapy with both ACE inhibitors and ARB were 10.8% of the total cohort.

There were a significantly lower percentage of subjects on ACE inhibitors and ACE inhibitors combined with ARBs in stage 4 than in stage 3 CKD patients (Table 3). No significant differences were noted among the percentage of patients on ARBs and combination therapy with ACEI and ARBs between stages 3 and 4 CKD. A slightly higher percentage of patients were on diuretics in stage 4 than stage 3. No significant differences were found in the percentage of patients on calcium channel blockers, beta blockers and alpha blockers between both the groups.

Hyperkalemia defined as potassium levels > 5.4 mEq/dl was more frequently observed in stage 4 than in stage 3 CKD (Table 3).

Proteinuria (> 300 mg/day) was present in 62.8% of the subjects with a mean of 1.2 ± 1.8 g/24 hrs) despite of the widespread use of ACE-inhibitors and ARB drugs. The mean protein excretion was not significantly different between stages 3 and 4 CKD groups (1.2 ± 1.8 vs. 1.3 ± 1.8 g/24 hrs). The percentage of patients with proteinuria however, was significantly higher in subjects with CKD stage 4 than in CKD stage 3 (66.8 vs 56.6%, p = 0.001).

The plasma lipid levels are given in Table 4. There were no significant differences in the LDL-cholesterol, HDL-cholesterol and the triglyceride levels in CKD stage 3 and 4 patients. The percentage of patients treated with statins was 54.7% and there were no differences in stage 3 and stage 4 patients (Table 4). Only one third of the patients had LDL-c below 100 mg/dl, and this percentage was lower for LDL-c < 70 mg/dl (8.3%). The percentage of patients treated with aspirin or other antiplatelet agents was 46.2% and no differences were found between stages 3 and 4 CKD patients.

**Table 4:** Lipids, statins and anti-platelet therapy

Characteristics	Total (n = 1129)	CKD Stage 3	CKD Stage 4	*P *value
Total cholesterol (mg/dL; mean ± SD [range])	195 ± 42 [30-171]	193 ± 38 [93-313]	196 ± 43 [74-396]	0.27

LDL-cholesterol (mg/dL; mean ± SD [range])	116 ± 37 [30-271]	116 ± 38 [39-217]	116 ± 36 [30-271]	0.94

HDL-cholesterol (mg/dL; mean ± SD [range])	50 ± 14 [2-99]	49 ± 13 [20-99]	51 ± 14 [23-99]	0.15

Triglycerides (mg/dL; mean ± SD [range])	147 ± 89 [40-900]	151 ± 92 [40-900]	143 ± 87 [40-860]	0.14

Patients treated with statins for cholesterol control (%)	54.7	56.2	53.2	0.30

Patients with LDL-c < 100 mg/dl (%)	34.8	32.9	36.0	0.32

Patients with LDL-c < 70 mg/dl (%)	8.3	7.6	8.8	0.52

Patients treated with aspirin or other anti-platelet agents (%)	46.2	45.3	47.11	0.52

Patients with serum albumin in normal range (%)	87.9	89.0	88.6	0.54

Serum albumin (g/dL) [range]	3.9 ± 0.4 [1.7-6]	3.9 ± 0.4 [1.7-6]	3.9 ± 0.4 [1.7-6]	0.79

### Anemia parameters and use of ESA and iron

According to the K-DOQI and the European Best Practices Guidelines (EBPG), anemia was present in 51.3% and 30.5% of the total subjects, respectively. The mean hemoglobin for the cohort as a whole was 12.8 g/dL and the majority of patients (87.8%) had hemoglobin greater than 11 g/dL (Table 5). The percentage of patients with severe anemia (hemoglobin < 9 g/dL) was 1.6% and the percentage of patients with hemoglobin between 9 and 11 g/dL was 11.5%. About 25% of the total cohort was receiving ESA and the mean hemoglobin in this group while receiving this therapy was 12 ± 1.5 g/dL and the range was wide (Table 5). The mean ferritin level was 150 ng/mL (range 5-1500) and the percentage of patients with transferrin saturation < 20% was 26.3%. The percentage of patients on oral and IV iron supplementation were 31.9% and 3.0%, respectively.

**Table 5:** Anemia parameters and the use of ESA and iron

Characteristics	Total (n = 1129)	CKD Stage 3 (*n *= 434)	CKD Stage 4 (*n *= 695)	*P *value
Anemia according to K-DOQI guidelines (%)	51.3	43.7	58.1	0.001

Anemia according to EBPG (%)	30.5	25.5	36.0	0.001

Hemoglobin (g/dL; mean ± SD [range])	12.8 ± 1.6 [7.6-18.7]	13.2 ± 1.7 [7.6-18.7]	12.4 ± 1.6 [7.6-16.9]	0.001

% of patients with Hemoglobin levels greater than 11 gm/dL	87.8	91.1	85.5	0.001

% of patients with Hemoglobin levels between 9 and 11 gm/dL	11.5	8.6	13.3	0.095

% of patients with Hemoglobin levels less than 9 gm/dL	1.6	0.5	2.3	0.001

Treatment with ESA* (%)	25.5	16.0	34.1	0.001

Mean hemoglobin levels in patients treated with ESA (g/dL; mean ± SD [range])	12.0 ± 1.5 [7.0-17.4]	12.3 ± 1.5 [9.3-17.4]	12.0 ± 1.5 [7.0-16.2]	0.15

Ferritin (ng/mL; mean ± SD [range])	150 ± 47 [5-1500]	129 ± 117 [5-997]	169 ± 168 [5-1500]	0.001

Iron (μg/dl; mean ± SD [range])	70 ± 26 [6-230]	75 ± 28 [6-230]	67 ± 25 [9-197]	0.001

Transferrin saturation < 20% (%)	26.3	23.4	28.5	0.12

Treatment with oral Iron (%)	31.9	23.6	39.5	0.001

Treatment with intravenous Iron (%)	3.0	1.6	4.4	0.001

The mean hemoglobin was significantly lower in stage 4 when compared to stage 3 CKD patients (12.4 ± 1.6 vs. 13.2 ± 1.7 g/dL). The percentage of patients with hemoglobin greater than 11 g/dL was significantly higher in stage 3 than stage 4 CKD patients (91.1 vs 85.5% p < 0.001). Conversely, the percentage of patients with hemoglobin < 9 g/dL was significantly lower in stage 3 than stage 4 CKD patients (0.5 vs 2.3% p < 0.001). No significant differences were noted in the percentage of patients with hemoglobin between 9 and 11 g/dL between CKD stage 3 and 4 patients (Table 5).

A significantly higher proportion of CKD stage 4 subjects were receiving ESA as compared to stage 3 CKD (34.1% vs. 16%, respectively). No significant differences were found in the mean hemoglobin between CKD stage 4 and 3 patients treated with ESA. The percentage of patients receiving ESA with hemoglobin levels < 9 g/dL, between 9-11 g/dL and > 11 g/dL were not significantly different in stages 3 and 4 CKD (Table 5). No significant differences were reported in the mean weekly dose of rHu-EPO and Darbepoetin in both the CKD groups (data not shown). No significant differences were noted among the patients with transferrin levels < 20% in both the groups. A significantly higher percentage of patients in CKD stage 4 were receiving oral and IV iron supplementation when compared to stage 3 CKD patients (39.5% vs. 23.6% and 4.4% vs. 1.6%, respectively) (Table 5).

**Figure 3:**
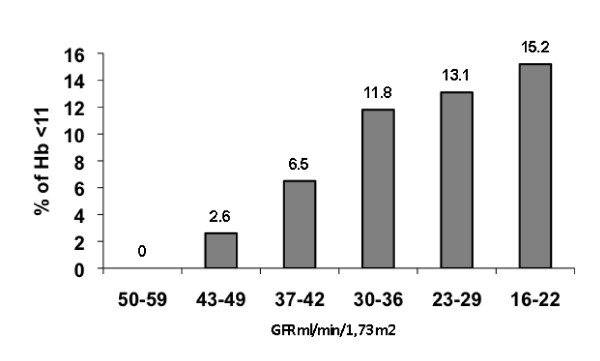
**Percentage of CKD stage 3 and 4 patients with hemoglobin less than 11 as a function of GFR**.

Figure 3 shows the distribution of anemia arbitrarily defined as Hb levels < 11 as a function of declining GFR. With declining GFR the % of patients with Hb < 11 g/dL increases progressively.

### PTH and calcium-phosphorus parameters

The mean phosphorus level was 3.7 mg/dL, the mean calcium-phosphorus product was 35 mg^2^/dL^2 ^and the mean intact PTH level was 145 pg/mL. The serum phosphorus was significantly higher in stage 4 than stage 3 CKD while the serum calcium was not significantly different (Table 6). The Ca-P product was slightly but significantly higher in stage 4 than stage 3. Intact PTH was significantly higher in stage 4 than stage 3 (166 vs. 121 pg/mL, p = 0.001).

**Table 6:** PTH and calcium-phosphorus metabolism.

Characteristics	Total (n = 1129)	CKD Stage 3 (*n *= 434)	CKD Stage 4 (*n *= 695)	*P *value
Phosphorus (mg/dL; mean ± SD [range])	3.7 ± 0.7 [1.7-6.7]	3.5 ± 0.6 [1.7-6.2]	3.8 ± 0.7 [1.7-6.7]	0.001

Serum phosphous within the K-DOQI target (2.8-4.6 mg/dL) (%)	85.1	86.8	83.5	0.12

Corrected calcium (mg/dL; mean ± SD [range])	9.3 ± 0.5 [7-11.8]	9.3 ± 0.5 [7.9-11.6]	9.3 ± 0.5 [7-11.8]	0.64

Serum calcium within the K-DOQI target (8.4-9.5 md/dL) (%)	57.6	57.5	57.6	0.99

Calcium-Phosphorus product (mg/dL, mean[SD] [Range]	35 ± 6 [14-65]	33 ± 6 [17-59]	36 ± 7 [14-65]	0.002

Percentage of patients with calcium levels below normal (< 8.4 mg/dl)	3.3	2.1	4.0	NS

Percentage of patients with phosphorus levels above normal	9.4	2.6	13.6	0.001

Calcium-Phosphorus product within the K-DOQI target (< 55 mg^2^/dL^2^) (%)	98.6	99.5	98.1	0.047

i-PTH (pg/mL; mean ± SD [range])	145 ± 116 [0-1166]	121 ± 99 [0-468]	166 ± 125 [9-1166]	0.001

Serum i-PTH within the K-DOQI target (%) [35-70 pg/mL (stage 3) or 70-110 pg/mL (stage 4)]	24.4	28.7	20.7	0.003

Meeting all four parameters according to K-DOQI guidelines (%)	10.5	12.2	9.0	0.10

Treatment with vitamin D (ergocalciferol/calcitriol) (%)	15.0	10.6	18.9	0.001

Treatment with phosphate binders (%)	19.7	11.9	26.7	0.001

85.1% of the patients had phosphorus levels within the K-DOQI target (Table 6) and 57.6% of the patients had mean calcium values within the K-DOQI target. 98.6% of the patients had the Ca-P product within the K-DOQI target whereas only 24.4% of the patients had mean intact PTH values within the K-DOQI target. The composite target for all the four parameters as per the K-DOQI guidelines was met in only 10.5% of the total cohort and 12.2 and 9.0% for stages 3 and 4, respectively.

The percentage of patients being treated with vitamin D supplementation and phosphate binders were 15% and 19.7%, respectively. A significantly higher percentage of patients were on vitamin D and phosphate binders in stage 4 than in stage 3 CKD (Table 6).

## Discussion

The MERENA study was designed as a prospective observational multicenter study of morbidity and mortality to characterize the delivery of care and outcomes after a 5 year follow up of CKD stage 3 and 4 patients under the care of nephrologists working in a network of hospitals of the Spanish National Healthcare System. Here we report the baseline characteristics of this cohort that includes 1,129 patients and discuss the most relevant differences between stage 3 and 4 CKD patients not only in terms of cardiovascular morbidity but also in the areas of control of hypertension and proteinuria, anemia management and mineral metabolism.

Individuals with reduced GFR are at a high risk of cardiovascular outcomes [6-11]. A recent study by Weiner et al. [9] confirmed that reduced GFR below 60 ml/min represents a risk factor for cardiovascular morbidity and mortality. In our study, GFR was estimated by both MDRD and Cockcroft-Gault formulas. Concordant with the findings of Weiner et al [9], and previous findings by others [6] our study found that reduced GFR below 59 ml/min, which includes CKD stages 3 and 4, was associated with a high prevalence of cardiovascular disease. However, the differences in CVD prevalence between stages 3 and 4 CKD were relatively small (35% vs. 42% in stages 3 and 4, respectively). To further analyze the calculated GFR effect on CVD, stage 3 subjects were subdivided into stage 3a and 3b. This analysis was done using not only the MDRD formula, which was the initial purpose of this study, but also using the Cockcroft-Gault formula and the more recently described EPI- CKD formula [19]. With all formulas CVD prevalence adjusted by age increases with declining GFR with the hierarchy (stage 3a < stage 3b < stage 4) (Figure 1). Of note, however, the change in CVD morbidity did not reach statistical significance with the MDRD formula. While the MDRD formula has been very useful to assess the burden of CKD in epidemiologic studies [6], it is worthy of comment that in our population the other two formulas appear to discriminate better regarding CVD than the MDRD formula.

The prevalence of CVD in stage 3 subjects older than 60 years in this Spanish cohort is similar than the 36.6% reported in CKD stage 3 subjects also older than 60 years in the USA by the National Health and Nutrition Examination Survey Study (NHANES) [20]. The prevalence of CVD in this Spanish cohort however, is higher than that reported from Italy in a cohort of patients managed by nephrologists in renal clinics (29.7%) [15]. In both studies from two Mediterranean countries where the delivery of care for CKD has similarities in terms of being provided by nephrologists relatively early in the course of the disease and under the auspices of a government funded system.

Among the traditional risk factors for CVD, current smoking was reported in 9.6% of the cohort and 10.5 and 8.8% of CKD stages 3 and 4, respectively. This is a prevalence of current smokers lower than that reported in the CRIC study, where smokers represented 14% of the total cohort [16]. Former smokers in our cohort were 36.2%. Among other traditional risk factors for CKD our patients were predominantly male and an overwhelming majority had hypertension (92.7%) and diabetes was present in 40.8% of the subjects. In the CRIC study, male preponderance was also noted (54%) but the data is not completely comparable because in this study the goal was to have equal gender for study selection purposes. Proteinuria was present in a majority of patients (62%) and was significantly higher in stage 4 than stage 3 CKD (See below).

The presence of hypertension in our cohort was almost universal (92.7%) without significant differences between stages 3 and 4 CKD (Table 3). The K/DOQI guideline recommended target of systolic < 130 and diastolic < 80 mm Hg [21] was achieved in only 17% of patients, less than that reported in the CRIC study (53%). In terms of control of hypertension, our data is only slightly better than the findings from a similar CKD cohort from Italy where only 12% of patients achieved BP of less than 130/80 mmHg [15]. It should be noted that the target blood pressure of < 130/80 mmHg may need to be re-evaluated in view of the recent findings that there were no significant differences in kidney disease progression between standard and intensive blood pressure control in the AASK study [22]. The mean systolic BP in our cohort was 141 ± 19 mm Hg and there were no significant differences between stages 3 and 4 CKD patients. As a group, this blood pressure is similar to the standard control-group in the AASK study. Nevertheless, when we consider a blood pressure target of 140/90 mmHg, 40.8% of the patients were on target (45.9% in CKD stage 3 vs 37.7% in CKD stage 4 (p = 0.007)). Although there is some debate, many experts think that there is little evidence among patients with CKD that a BP goal of < 130/80 mmHg saves lives, saves kidneys or reduces cardiovascular events [23]. Hypertension therapy should be individualized using home BP monitoring and the precise targets must await randomized controlled trials [23].

A large percentage of patients were receiving an ACE inhibitor (43.3%), an ARB (44.8%) or combined ACE inhibitors and ARBs (10.8%). The use of these agents was significantly higher in stage 3 than in stage 4 CKD patients (81.9 vs. 72.4%). The percentage of CKD 4 patients receiving diuretics was higher in stage 4 than stage 3 CKD patients. There were no other significant differences between stage 3 and 4 CKD patients regarding other anti hypertensive agents. Plasma potassium was slightly but significantly higher in stage 4 than stage 3 CKD patients and as expected the percentage of patients with hyperkalemia was higher in stage 4 than in stage 3 CKD (17.1 and 9.8%, respectively p < 0.001).

The data in our study regarding proteinuria was similar to that reported in an Italian CKD cohort where the mean protein excretion was 0.8 and 1.2 g/24 hrs in stages 3 and 4, respectively. By contrast the degree of proteinuria in CRIC study was much lower (0.17 g/24 hours). The most likely explanation for the lesser degree of proteinuria may be related to the better BP control in the CRIC study where over 50% of the patients had achieved a BP < 130/80 mm Hg. Consistent with this notion the recently published AASK study showed improved control of proteinuria with intensive blood pressure control [22].

The prevalence of anemia and its severity was higher in CKD stage 4 than CKD stage 3 patients and a larger proportion of CKD stage 4 than stage 3 patients were receiving ESAs. In those receiving ESAs, there were no differences in the mean weekly dose between stages 3 and 4 CKD (Table 5). Of note, the mean hemoglobin of the cohort was relatively high (12.8 g/dL) and the majority of the patients had hemoglobin greater than 11 g/dL (87.6%). Since only 25.5% of the patients in the total cohort were receiving ESA therapy, the relatively high hemoglobin of the group as a whole cannot be attributed to overtreatment with these agents. As expected the prevalence of anemia, defined arbitrarily as Hb < 11 g/dL, increased as the estimated GFR declines (Figure 3).

About one third of the patients were receiving iron therapies, mostly in the form of oral iron supplements. Transferrin saturation < 20% was documented in 26.3% of the patients therefore, suggesting under treatment with iron in two significant portions of patients. These findings suggest that there is a need for further utilization and/or optimization of iron therapies in the CKD patients.

We think that the information from our cohort has implications for the management of CKD associated anemia. The initiation of ESA and maintenance of its use in these patients has been generally targeted according to the K-DOQI guidelines [24]. In our cohort, the mean hemoglobin was 10.3 g/dL during the initiation of ESA therapy. The use of ESA has been under great scrutiny and a moving target [24-27]. There is now a need to reevaluate the management of anemia in CKD and the data from this study will be timely. Clearly, our findings suggest that the lower targets for hemoglobin that are currently being proposed are achievable with reduced use of ESAs in many CKD stages 3 and 4 patients.

Regarding calcium-phosphorus and PTH levels, a salient finding is that only 28.7% of subjects with stage 3 and 20.7% of subjects with stage 4 had levels of PTH within the recommended targets based on K-DOQI guidelines [28-31]. On the other hand, the majority of subjects had phosphorus levels within the K-DOQI guidelines and this was also reflected in the Ca-P product within the K-DOQI guidelines in the majority of subjects with both stage 3 and 4 CKD. This was accomplished even though only 11.9 and 26.9% of the subjects were receiving phosphate binders in CKD stages 3 and 4, respectively. This probably reflects that an increase in serum phosphorus occurs with more advanced CKD. There was also very low percentage of subjects receiving vitamin D and its analogues and this explains that the levels of PTH were not in the desired target in the majority of patients with CKD stages 3 and 4. Therefore, it is reasonable to conclude that the overall management of secondary hyperparathyroidism in this cohort could benefit from treatment with Vitamin D and its analogues.

## Conclusions

In summary, the baseline characteristics of the MERENA cohort provide information regarding a high level of cardiovascular morbidity in stages 3 and 4 CKD patients with a gradual increase noted when stage 3 is subdivided into stages 3a and 3b. The presence of hypertension was almost universal in stages 3 and 4 CKD despite the use of more than 3 anti-hypertensive agents including widespread use of RAS blockers. The majority of patients had secondary hyperparathyroidism despite reasonable control of calcium-phosphorus levels suggesting a need for vitamin D based therapies. Finally, a majority of the patients had hemoglobin levels greater than 11 g/dL while the use of ESA was limited to about 25% of patients.

In conclusion, this study provides an overview of key clinical parameters in patients with CKD Stages 3 and 4 where delivery or care was largely by nephrologists working in a network of hospital-based clinics of the Spanish National Healthcare System.

## Competing interests

The authors declare that they have no competing interests.

## Authors' contributions

AMC, JLG, JP, FA, AC, JL, JFN, RM have participated on the protocol designed, drafted the manuscript, coordination of the study in the different regions and they are the members of the scientific committee of the study. AMC, JLG, DB and AN have participated on the analysis of the available data and preparation of the manuscript after the draft suggested by the previous authors. JJDC and JLG have performed the statistical analysis. AC, JFN, FdA and RM have participated as principal investigators as well as in the corrections of the final draft of the manuscript. All authors read and approved the final manuscript

## Pre-publication history

The pre-publication history for this paper can be accessed here:

http://www.biomedcentral.com/1471-2369/12/53/prepub
